# Platelet Activating Factor Contributes to Vascular Leak in Acute Dengue Infection

**DOI:** 10.1371/journal.pntd.0003459

**Published:** 2015-02-03

**Authors:** Chandima Jeewandara, Laksiri Gomes, N. Wickramasinghe, Danuta Gutowska-Owsiak, Dominic Waithe, S. A. Paranavitane, N. L. A. Shyamali, Graham S. Ogg, Gathsaurie Neelika Malavige

**Affiliations:** 1 Centre for Dengue Research, University of Sri Jayawardanapura, Nugegoda, Sri Lanka; 2 MRC Human Immunology Unit, NIHR Biomedical Research Centre, Weatherall Institute of Molecular Medicine, Oxford, United Kingdom; 3 Department of Medicine, Faculty of Medical Sciences, University of Sri Jayawardanapura, Nugegoda, Sri Lanka; University of North Carolina at Chapel Hill, UNITED STATES

## Abstract

**Background:**

Although plasma leakage is the hallmark of severe dengue infections, the factors that cause increased vascular permeability have not been identified. As platelet activating factor (PAF) is associated with an increase in vascular permeability in other diseases, we set out to investigate its role in acute dengue infection.

**Materials and Methods:**

PAF levels were initially assessed in 25 patients with acute dengue infection to determine if they were increased in acute dengue. For investigation of the kinetics of PAF, serial PAF values were assessed in 36 patients. The effect of dengue serum on tight junction protein ZO-1 was determined by using human endothelial cell lines (HUVECs). The effect of dengue serum on and trans-endothelial resistance (TEER) was also measured on HUVECs.

**Results:**

PAF levels were significantly higher in patients with acute dengue (n = 25; p = 0.001) when compared to healthy individuals (n = 12). In further investigation of the kinetics of PAF in serial blood samples of patients (n = 36), PAF levels rose just before the onset of the critical phase. PAF levels were significantly higher in patients with evidence of vascular leak throughout the course of the illness when compared to those with milder disease. Serum from patients with dengue significantly down-regulated expression of tight junction protein, ZO-1 (p = 0.004), HUVECs. This was significantly inhibited (p = 0.004) by use of a PAF receptor (PAFR) blocker. Serum from dengue patients also significantly reduced TEER and this reduction was also significantly (p = 0.02) inhibited by prior incubation with the PAFR blocker.

**Conclusion:**

Our results suggest the PAF is likely to be playing a significant role in inducing vascular leak in acute dengue infection which offers a potential target for therapeutic intervention.

## Author Summary

Although plasma leakage is the hallmark of severe dengue infections, the factors that cause increased vascular permeability have not been identified. As platelet activating factor (PAF) is associated with an increase in vascular permeability in other diseases, we set out to investigate its role in acute dengue infection. In this study, we found that PAF was significantly increased in patients with DHF, and the PAF levels rose just before the onset of the critical phase of dengue, during which vascular leak is thought to occur. PAF in serum of dengue patients was associated with reduced expression of tight junction proteins (ZO-1) and reduction in trans-endothelial resistance (TEER) of human endothelial cells. Use of PAFR blockers significantly reduced the down regulation of ZO-1 by serum of dengue patients and also the reduction of TEER, suggesting that PAF plays a significant role in inducing vascular leak in acute dengue infections.

## Introduction

Dengue is thought to infect 390 million individuals per year resulting in approximately 96 million clinically apparent infections[1]. The annual burden of dengue has been estimated to be 750,000 disability adjusted life years (DALYs)[2] which is higher than the global burden of 17 other disease conditions, including upper respiratory tract infections, hepatitis and Japanese Encephalitis[3]. It has been declared a priority infection by the WHO, UNICEF and World Bank[4]. Currently there are no effective antiviral drugs to treat acute infection, nor a licensed vaccine to prevent infection.

Dengue infections are caused by four dengue virus (DENV) serotypes that are highly homologous [5]. Infection with any one of these serotypes can lead to asymptomatic infection disease or may manifest as undifferentiated viral fever, dengue fever or result in severe dengue infections in the form of dengue haemarrohagic fever (DHF), dengue shock syndrome (DSS) or expanded syndrome of dengue infection. Expanded syndrome of dengue infection is characterized by isolated organ involvement such as liver failure, myocarditic or encephalitis [4]. Although the majority of infections are asymptomatic or cause mild clinical disease, DHF and DSS are associated with a high morbidity and often with fatal outcomes. Increased vascular permeability leading to vascular leak is the hallmark of severe dengue infection [6]. Although the exact timing of vascular leak is not fully known, it is thought to occur early during infection and then substantially increase during the critical phase when it can be detected clinically or by laboratory methods [7]. The critical phase of dengue infection is thought to last for 24 to 48 hours following which the leaked fluid is reabsorbed and the patient recovers [4]. Complications as a result of plasma leakage such as shock, pleural effusions, ascites along with other complications such as liver failure and encephalopathy, also occur during the critical phase [4]. Currently the causes of increased vascular permeability are unknown. However, due to the rapid reversibility of increased vascular permeability, endothelial dysfunction rather than necrosis of the endothelium is thought to be the cause of vascular leak [6]. In fact, in postmortem studies, neither dengue NS1 antigen, viral protein or complement components have been detected in the endothelium, suggesting that endothelium dysfunction is likely mediated by host inflammatory mediators [8].

Cytokines and other mediators such as VEGF, TNFα and MCP-1 have been suggested to contribute to endothelial dysfunction and lead to vascular leak in dengue [9–14]; among these, VEGF has been extensively studied[12,13] and it has been documented that plasma VEGF levels correlated with vascular leak [13]. Using Human Endothelial cell lines (HUVECs) Appana *et al.* have shown that factors causing vascular leak are likely to be present in serum of dengue patients. In their experiments, sera from patients with acute dengue have shown to reduce expression of gap junction proteins and disrupt morphology of HUVECs [15]. Apart from the above, lipid mediators such as platelet activating factor (PAF), are known to play a role in increasing vascular permeability in disease conditions such as sepsis and anaphylaxis [16–19]. PAF is believed to be essential for the increase in vascular permeability and associated inflammatory changes seen in cerebral malaria [20]. PAFR^−/−^ mice have shown to be less susceptible in developing severe dengue than the wild type mice [21]. In addition, thrombocytopenia and haemoconcentration observed in the wild type mice was significantly reversed by use of a PAFR blocker [21]. Platelets have been shown to be highly activated in dengue and platelet-monocyte aggregates were found to correlate with thrombocytopenia and increased vascular permeability [22]. Activation of both platelets and complement and release of inflammatory mediators is proposed as an alternate mechanism that causes vasculopathy leading to the plasma leakage [23].

The role of lipid mediators such as PAF has not been studied in dengue infection in humans. However, as PAF is involved in vascular leak in other diseases such as sepsis and anaphylaxis and since there is evidence of its potential role in causing vascular leak in mouse models, it would be crucial to evaluate the role of PAF in triggering vascular leak in acute dengue infection. In this study we found that PAF was significantly increased in patients with DHF and that the PAF levels rose just before the onset of the critical phase of dengue, during which vascular leak is thought to occur. PAF in serum of dengue patients altered expression pattern of tight junction protein ZO-1 and decreased the integrity of human endothelial cell monolayer, as measured by trans-endothelial resistance (TEER). Prior use of PAFR blocker significantly reduced these effects, suggesting that PAF plays a significant role in inducing vascular leak in acute dengue infection.

## Materials and Methods

In the initial phase of the study, 25 adult patients with clinical features suggestive of dengue infection, admitted to a general medical ward in a tertiary care hospital (Colombo South Teaching Hospital) in Colombo during the year 2013, were enrolled following informed written consent. 16 of these patients developed DHF and 9 had DF. 12 dengue-seropositive healthy individuals were also recruited for the initial assays of lipid mediators.

Another 36 adult patients were enrolled for the second phase of the study, in which serial blood samples were taken in the morning (6 a.m.) and at 1.00p.m., from the time of admission to the time of discharge from hospital. The onset of illness was defined as the time of onset of fever. If the patient was recruited following 3 days of fever, it was considered as the duration of illness to be 72 hours. As patients with DHF were in hospital for a longer time than those with DF, approximately 5 to 7 serial blood samples were collected from them, but only 3 to 4 samples were collected from those with DF.

### Ethics statement

The study was approved by the Ethics Review Committee of the University of Sri Jayawardanapura. All adult patients provided informed written consent.

All clinical features, such as presence of fever, abdominal pain, vomiting, bleeding manifestations, hepatomegaly, blood pressure, pulse pressure and evidence of fluid leakage were recorded several times each day. The full blood counts, the alanine transaminase (ALT) and aspartate transaminase (ALT) levels were assessed during the course of the illness. Clinical disease severity was classified according to the 2011 WHO dengue diagnostic criteria [4]. The classification of whether the patient had DF or DHF was decided by the attending physician at the time of discharge after carefully reviewing the clinical and laboratory features and complications. Accordingly, patients with a rise in haematocrit above ≥ 20% of the baseline haematocrit or clinical or ultrasound scan evidence of plasma leakage in a patient was classified as having DHF. Shock was defined as having cold clammy skin, along with a narrowing of pulse pressure of ≤ 20 mmHg. According to this definition 25 patients were diagnosed to have DHF and 11 DF. Although some patients had very low platelet counts <25,000 cells/mm^3^, and high liver enzymes, they were classified as having DF since there was no evidence of fluid leakage.

### Quantitative PAF, PAF-AH and PAFR assays

Quantitative PAF, PAF-acetyl hydrolase and secretory PAF receptor levels were done in duplicate on all serum samples by quantitative ELISA. Levels of PAF, PAF-acetyl hydrolase (PAF-AH) and PAF receptor levels (PAFR) were initially done in the 25 patient samples and also in 12 healthy dengue seropositive individuals to determine if PAF, PAF-AH and PAFR were different in patients and healthy individuals before carrying out these assays in serial serum samples. Following the initial assessment of PAF in the 25 patient samples, the PAF levels were done in duplicate in all 36 serial samples. The levels of PAF, PAF-AH and PAFR levels (Cusabio, China) were carried out according to manufacturers’ instructions.

### Serology

Acute dengue infection was confirmed in the serum samples using the NS1 early dengue ELISA (Panbio, Australia). All assays were done in duplicate. Dengue was also confirmed in these patients with a commercial capture-IgM and IgG enzyme-linked immunosorbent assay (ELISA) (Panbio, Brisbane, Australia). The ELISA was performed and the results were interpreted according to the manufacturers’ instructions. This ELISA assay has been validated as both sensitive and specific for primary and secondary dengue virus infections [24,25].

### HUVEC cell culture

Human umbilical vein endothelial cells (HUVECs) (Lonza, Switzerland) were maintained in endothelial cell–based medium 2 (Lonza, Switzerland) supplemented with 10% fetal calf serum and growth factors (human epidermal growth factor, hydrocortisone, human recombinant fibroblast growth factor-beta, vascular endothelial growth factor, Insulin-like growth factor, Ascorbic acid, Heparin, FBS, and Gentamicin/Amphotericin-B) at 37°C at 5% CO2. Cells were grown in culture flasks or culture slides (BD, USA) pre-coated with 0.1% gelatin (Sigma, UK). Pre-coating was carried out by incubating flasks with 100 uL/cm2 of 0.1% gelatin at 37°C for 2 hours. PAF and PAF receptor antagonist (1-(N,N-Dimethylcarbamoyl)-4-ethynyl-3-(3-fluoro-4-((1H-2-methylimidazo[4,5-c]pyridin-1-yl)methyl)benzoyl)-indole, HCl (Calbiochem, Germany), (both from Millipore, Germany) were used for treatments. Both PAF and PAFR blocker were diluted in dH_2_O according to the manufacture instructions and aliquoted. PAF was stored in - 20°Ca and PAFR antagonist was stored in 4°C until further use.

### Confocal imaging

Endothelial cells were seeded into gelatin-coated eight-well culture slides. The following day, serum samples from dengue patients (diluted in medium at a ratio of 1:3) or PAF were added and incubated for 3 hours at 37°C at 5% CO2. HUVECs were then immunostained for ZO-1 as described below. In experiments with PAF blockage, PAFR blocker was added to the culture medium one hour prior the addition of PAF or dengue serum.

### Immunofluorescence staining ZO-1

HUVEC cells grown in cell chambers (BD, USA) were fixed with 2% paraformaldehyde (Alfa Aesar, UK) for 10 minutes and permeabilized (0.1% Triton X-100; Sigma, UK) for five minutes at room temperature. The cells were then blocked with 2% BSA and 5% FCS for 45 minutes. Purified rabbit monoclonal anti-human ZO-1 antibody (Life technologies, USA) (1:200 dilution) and secondary Alexa Fluor 488 mouse anti-rabbit IgG (heavy and light chains) (Invitrogen, USA) or Alexa Fluor 568 mouse anti-rabbit IgG (heavy and light chains) were used for staining; NucBlue Live ReadyProbes (Molecular probes, USA) was used to stain nuclei. Cells were then mounted with Mowiol 4–88 fluorescent mounting medium (Sigma, UK), and the data was acquired on a Zeiss LSM 780 Confocal Inverted Microscope; the image analysis performed using Image J software 1.47v (NIH,USA ). Confocal images were analysed using an automated FIJI macro. Discrete ZO-1 staining was isolated from images by taking raw image and subtracting Gaussian smoothed (sigma = 15) duplicate of the image. To make segmentation easier of the background-subtracted images, a Gaussian kernel (sigma = 2.0) was then applied to remove high-frequency noise from the clusters. Images were then threshold and the resulting binary mask skeletonised using the Fiji ‘skeletonize’ function. The binary fragments were then quantified using the ‘Analyse Particles’ plugin and the area summed to give a measure of total tight junction expression per image. To give an expression level per cell, the area sum value per image was divided by the number of cell nuclei present, as indicated by DAPI nuclear staining. All imaging experiments done in triplicate and five image fields per condition were obtained to include in the analysis.

### Trans-endothelial electrical resistance (TEER) measurements

24 well tissue culture plates and cell inserts (BD, USA) were coated with 500μl and 200μl 0.1% gelatin, respectively, and incubated for 2 hours at 37°C before adding the cells. The gelatin was washed with PBS and cell inserts kept in a in a companion plate (353504, BD, USA) and EGM-2 media (Lonza, Switzerland). Both to the insert (200μl) and the companion plate (700μl) were washed before addition of cell suspensions at a concentration of 50,000 cells/50μl of media and cultured overnight at 37°C in 5% CO2. On the following day, the inserts were transferred in to another companion plate with 700μl of EGM-2/well and 250μl of EGM2 was added in the insert prior to measuring TEER using Millicell-ERs (Fisher Scientific, UK). The experiments were carried out when the HUVECs formed a confluent monolayer and plateau of electronic resistance was observed on the 3^rd^ day when the resistance reached 450 ohms. 10 different experiments were carried out in triplicate using serum from healthy individuals and also serum from healthy individuals with PAFR blocker (1-(N,N-Dimethylcarbamoyl)-4-ethynyl-3-(3-fluoro-4-((1H-2-methylimidazo[4,5-c]pyridin-1-yl)methyl)benzoyl)-indole, HCl (Calbiochem, Germany). To determine the effect of dengue sera on TEER, 9 separate experiments with dengue serum in the presence and absence of PAFR blocker were carried out in three biological replicates. In all the experiments the PAFR blocker was added 1 hour prior to adding serum of dengue patients and incubated at 37°C.

### Statistical analysis

Statistical analysis was performed using Graph Pad PRISM version 6. As the data were not normally distributed, differences in means were compared using the Mann-Whitney U test (two tailed); when three or more groups were compared Kruskal Wallis test was used. Receiver-operator characteristic (ROC) curves, showing the area under the curve (AUC) were generated to determine the discriminatory performance of the highest serum PAF level detected with regard to severity of illness.

## Results

**Figure 1:**
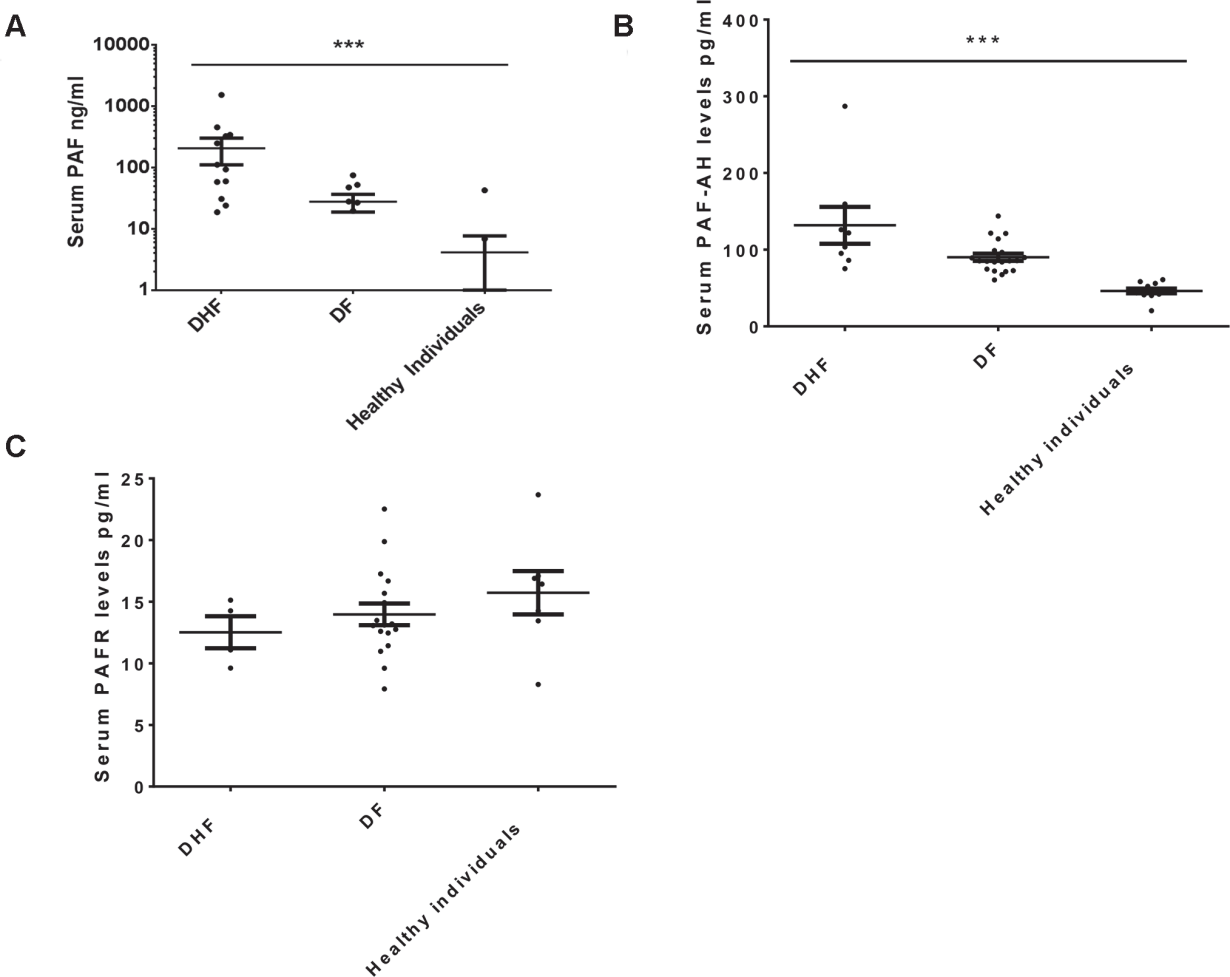
PAF, PAF-AH and PAF-R levels in patients and healthy individuals. A: PAF levels in sera of patients with DHF (n = 16), DF (n = 9) and healthy individuals (n = 12). The bars represent the mean and the standard error of mean; p = 0.002. B: PAF-AH levels in sera of patients with DHF (n = 16), DF (n = 9) and healthy individuals (n = 12). The bars represent the mean and the standard error of mean; p<0.0001. C: PAFR levels in sera of patients with DHF (n = 16), DF (n = 9) and healthy individuals (n = 12). The bars represent the mean and the standard error of mean; p = 0.33.

Serum samples for analysis of PAF, PAF-AH and soluble PAF-Receptor was obtained on day 5–6 of illness. In the initial analysis of all 25 patients with acute dengue infection and healthy individuals, PAF levels were significantly higher in patients (p = 0.002) when compared to healthy individuals. Although not significant (p = 0.15), PAF levels were higher in patients with DHF (median 335.2, Inter quartile range 4.7 to 443.1 ng/ml) when compared to those with DF (median 47.63, IQR 0 to 111.6 ng/ml) (Fig. 1A). Since high PAF values could be either due to increased production or reduced breakdown, we also analysed PAF—acetyl hydrolase (PAF-AH) levels in these patients. PAF-AH is the enzyme that breaks down PAF [26]. PAF-AH levels have been shown to be low in some diseases such as asthma and anaphylaxis and thus thought to contribute to disease pathogenesis by reduced breakdown of PAF [27,28]. We found that PAF-AH levels were significantly higher (p<0.0001) in patients with acute dengue when compared to healthy individuals. The PAF-AH levels were significantly higher (p = 0.01) in those with DHF (median 112.6, IQR 88.35 to 151.1 ng/ml) when compared to those with DF (median 85.35, IQR 73.2 to 98.3 ng/ml) (Fig. 1B) suggesting that high PAF values were not due to reduced breakdown of PAF. We also assessed soluble PAF-Receptor levels in serum and found that there was no difference in PAF-R levels in patients with acute dengue or in healthy individuals (Fig. 1C).

### Kinetics of PAF levels in patients with DHF and DF

**Figure 2:**
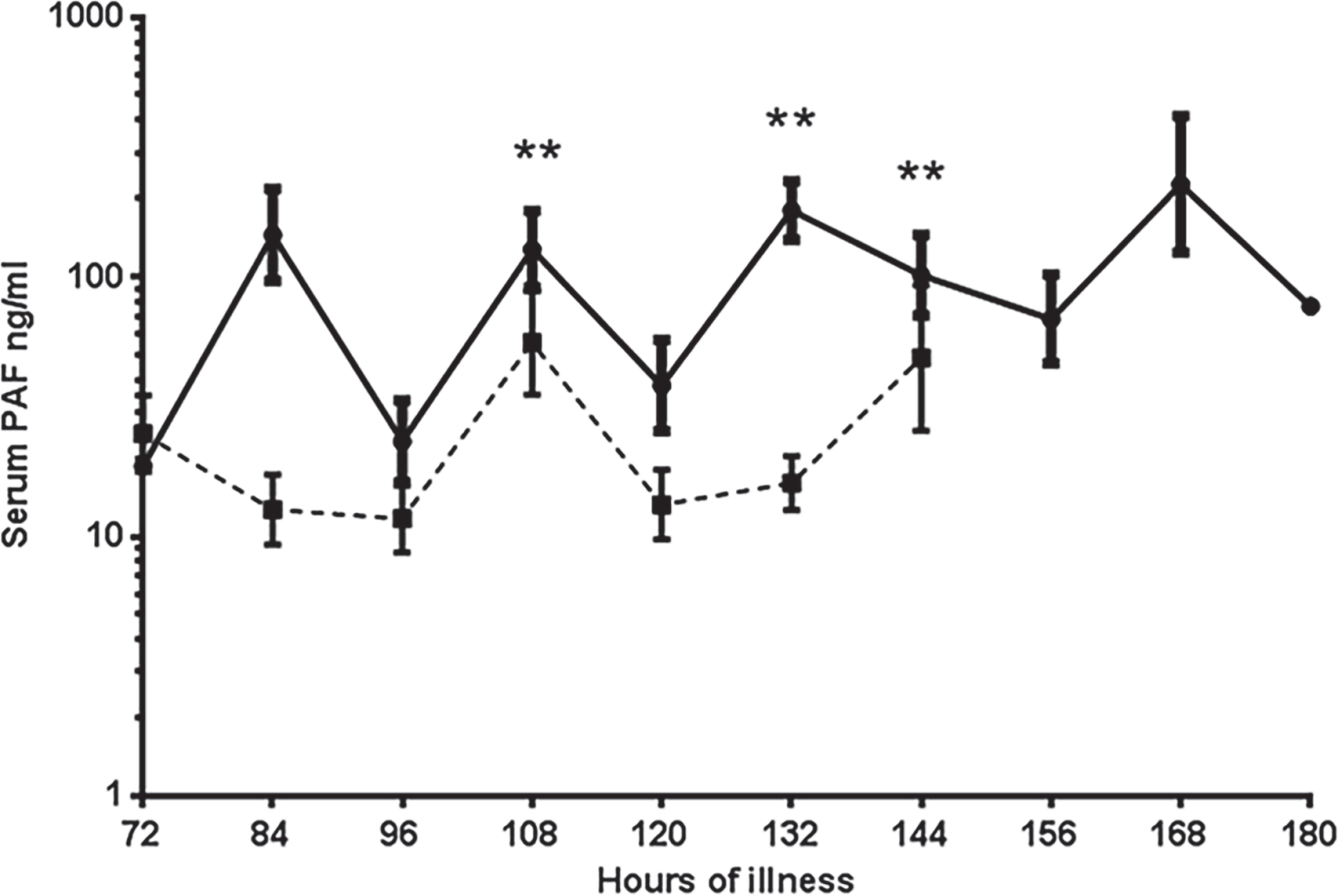
Kinetics of PAF. Levels of PAF were measured twice a day throughout the course of illness in patients with DF and DHF from the time of admission to the ward until time of discharge. ** indicate time points where the PAF values were significantly higher in patients with DHF when compared to those with DF.

As dengue infection is a very dynamic disease, the patients for determining kinetics of PAF were recruited on a mean of 106.2 (SD±19.2) hours of illness. As we found that PAF levels were significantly higher in patients with acute dengue, we assessed PAF levels in serial blood samples collected from patients throughout the course of the illness. Patients with DHF had significantly higher PAF throughout the course of the illness when compared to those with DF (Fig. 2). However, there was a wide variation in the PAF levels in patients from both groups. Except for 3 patients with DF the PAF levels of all other patients (8/11) with DF never rose to >100 ng/ml throughout the course of the illness. Of these 3 patients who had higher values, one patient had platelet counts that dropped to 30,000 cells/mm^3^ and she also complained of vaginal bleeding in the absence of her usual menstrual period. However, she was classified as having DF as she did not have any clinical or laboratory evidence of fluid leakage. The second patient with DF whose PAF levels rose to 293.16 ng/ml, also complained of vaginal bleeding in the absence of menstruation. The other DF patient whose PAF levels rose to 123.4 ng/ml only had a mild rise in liver enzymes, no evidence of fluid leakage and no bleeding manifestations. 3/25 patients with DHF had values <100 ng/ml. One of these patients presented to hospital on day 6 of illness and had already progressed to the critical phase. The other 2 patients were not in the critical phase on admission and were admitted to hospital on day 4.

Interestingly, a diurnal variation in PAF levels was observed in the majority of patients with DHF but not in those with DF.

### The effect of dengue sera on ZO-1 expression on HUVECs

**Figure 3:**
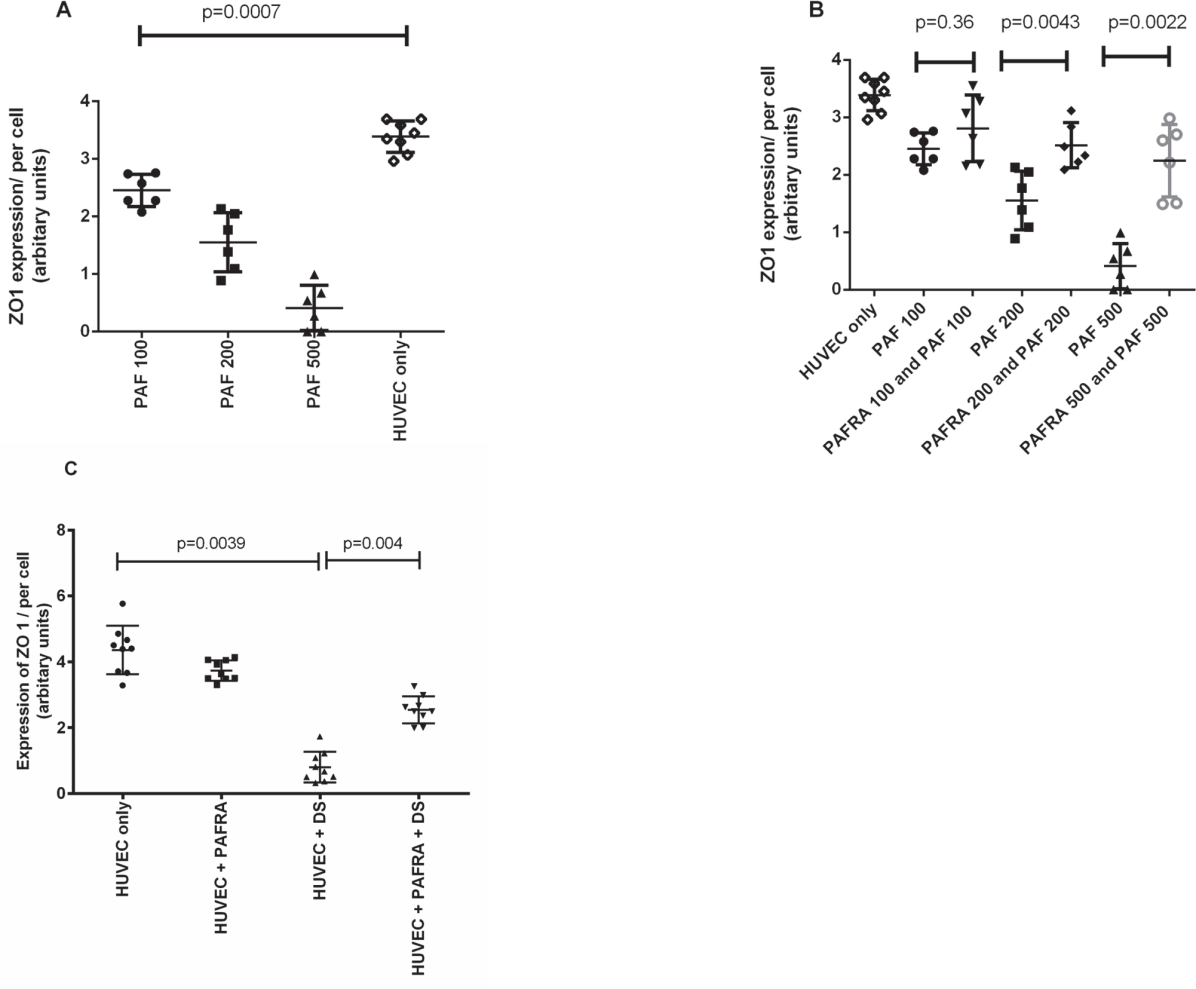
Effect of PAF and PAFR blocker on expression of ZO-1. A: ZO-1 expression in HUVECs was evaluated with different concentrations of PAF. ZO-1 expression was compared in untreated HUVECs, with HUVECs incubated with 100ng/ml PAF; 200ng/ml PAF and 500ng/ml PAF. B: The differences in ZO-1 expression were evaluated in HUVECs treated with different concentrations of PAF. The HUEVECs were pretreated with a PAFR blocker for one hour prior to been treated with different concentrations of PAF. C: ZO-1 expression was evaluated in HUVECs that were treated with media alone (untreated) compared to HUVECs treated with 500ng/ml PAFR blocker alone; treated with dengue patient serum (DS) and pre-treated with a PAFR blocker (PAFRA) prior to treatment with dengue patient serum (DS)

As PAF has shown to reduce expression of ZO-1 in HUVECs and increase endothelial permeability, we initially assessed if similar observations were found in our model. As expected, the use of PAF on HUVECs significantly reduced (p = 0.007) surface expression of ZO-1, which was dose dependent (Fig. 3A). ZO-1 expression of HUVECs was significantly up regulated in a dose dependant manner with the use of a PAFR blocker (Fig. 3B). The PAFR blocker(1-(N,N-Dimethylcarbamoyl)-4-ethynyl-3-(3-fluoro-4-((1H-2-methylimidazo[4,5-c]pyridin-1-yl)methyl)benzoyl)-indole, HCl (Calbiochem, Germany), potentially inhibits binding of PAF to its receptor in a competitive manner in equilibrium binding studies. A non-competitive inhibition is reported if the membrane bound PAFR are pre-incubated with this blocker before adding PAF, which is thought to be due to a slower antagonist dissociation rate (Calbiochem, Germany). Since the inhibition of PAF was most significant when the PAFR blocker was used at a concentration of 500ng/ml, this concentration was used for other blocking experiments.

**Figure 4:**
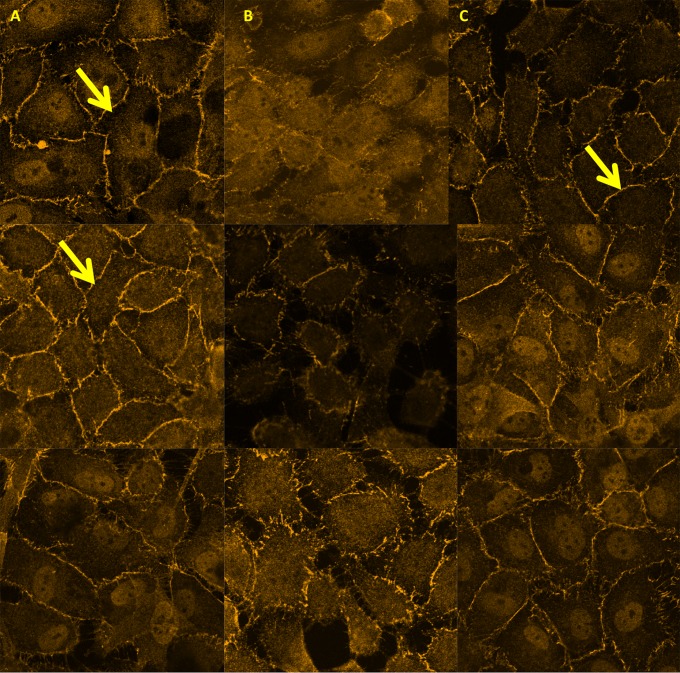
Immunofluorescence analysis of tight junction protein ZO-1 on HUVECs. Images of HUVECs incubated with EGM, dengue serum and pre-treated with a PAFR blocker prior to addition of dengue serum from 3 patients are shown. Cells were treated with A – Endothelial growth medium only; B- treated with dengue patient serum (n = 3); C- HUVEC pre-treated a PAFR blocker before addition of dengue serum (n = 3. All assays were done in duplicate and repeated twice. Five separate images per condition were analysed quantitatively. Arrows indicate the ZO-1 staining.

The above experiments showed that PAF reduces expression of ZO-1 and this effect was significantly inhibited by the use of a PAFR blocker, we then proceeded to determine the effect of serum from dengue patients on expression of ZO-1. We also found that serum from patients with DHF significantly downregulated ZO-1 expression (p = 0.004) (Fig. 3C and Fig. 4). Furthermore, HUVEC cells incubated with serum from DHF patients showed disrupted morphology, reduced ZO-1 expression and widening of gap junctions (Fig. 4). However, the down regulation of ZO-1 expression by dengue sera was significantly inhibited (p = 0.004) by incubating HUVECs with a PAFR blocker (Fig. 3C and Fig. 4).

The HUVECs incubated with serum from dengue patients showed disrupted morphology, reduced ZO-1 expression and widening of gap junctions (Fig. 4). The ZO-1 expression was seen to be restored when HUVECs were pre-incubated with a PAFR antagonist (Fig. 4).

### Effect of dengue sera on trans-endothelial resistance

**Figure 5:**
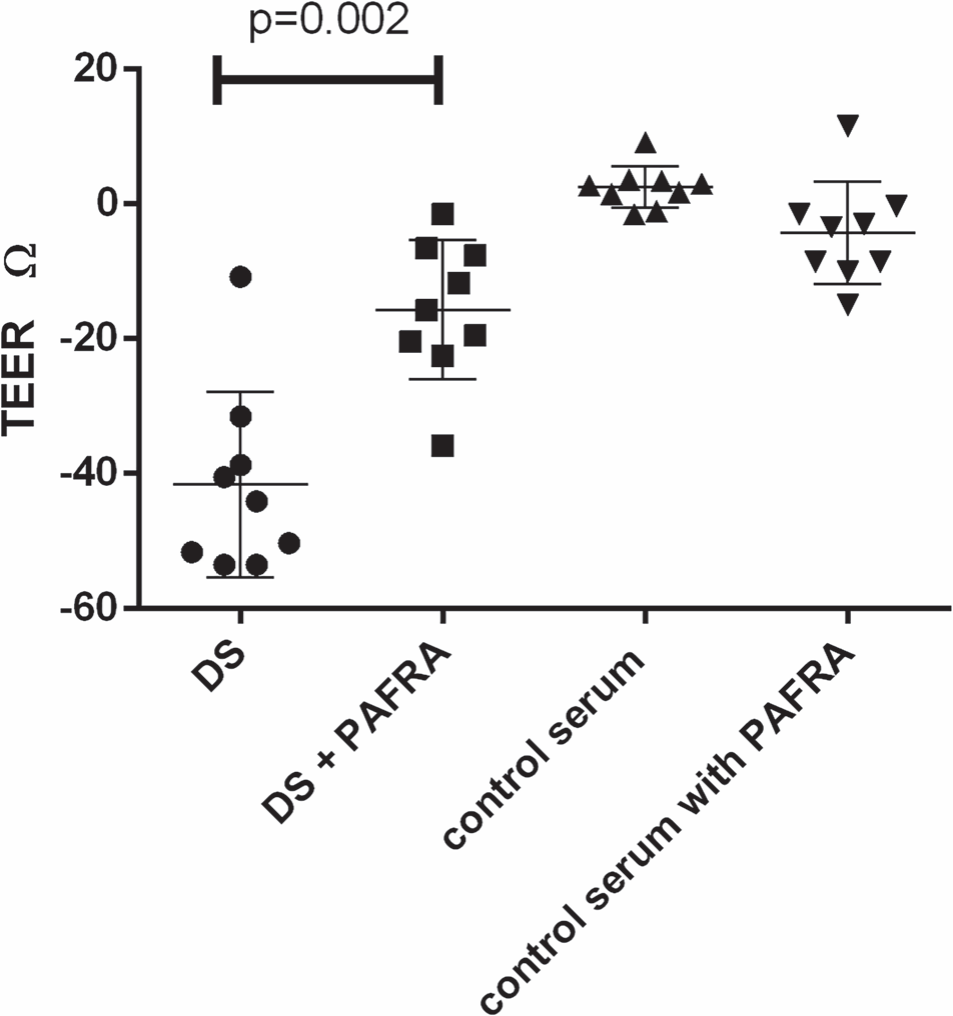
Changed in the trans-endothelial electrical resistance with dengue sera and the effect of PAFR blocker. Change in TEER with dengue serum (DS; n = 9); with DS following pretreatment with a PAFR blocker (PAFRA n = 9); with control serum (n = 9); with in control serum following pretreatment with a PAFR blocker (n = 9).

As the above experiments showed that both dengue serum and PAF affected on ZO-1 expressionin a dose dependant manner, which was inhibited by the use of a PAFR blocker, and also the effect of dengue serum of ZO-1 expression was inhibited by a PAFR blocker, we next proceeded to determine the effect of dengue sera on trans-endothelial resistance (TEER). The use of serum from dengue patients significantly reduced TEER (mean −35.82, SD ± 12.93 Ώ) when compared to use of serum from healthy individuals (mean 1.96, SD ± 1.88 Ώ). This reduction in TEER by dengue serum was significantly (p = 0.002) inhibited by the use of a PAFR blocker prior to incubation of the HUVECs with dengue sera (Fig. 5).

## Discussion

In this study we have investigated the role of PAF as a mediator of vascular leak in acute dengue infection. We found that PAF levels were significantly elevated in patients with dengue infection, as well as its principle breakdown enzyme PAF-AH, which suggested that the increase in PAF was likely due to increased production rather than reduced breakdown. We also found that PAF levels were significantly higher in patients with DHF throughout the course of the acute disease when compared to those with DF, although huge inter-individual variations in PAF levels were observed. PAF has been shown to be important in vascular leak in dengue mice models and PAFR^−/−^ mice were shown to have milder clinical disease [21]. The role of PAF in human dengue infection has only been investigated in the context of *in vitro* studies where mononuclear leucocytes of dengue immune donors were found to produce more PAF than non-immune donors [29].

HUVECs models have been widely used to assess increase in vascular permeability by many mediators and drug molecules as well as to determine changes in trans-endothelial electrical resistance (TEER) [11,15,30]. Experiments carried out by Appanan *et al.* showed that mediators present in the serum of dengue patients reduced expression of tight junction and adherent junction proteins which are likely to result in increased vascular permeability [15]. We found that PAF reduces expression of ZO-1 in a dose dependent manner and this downregulation of ZO-1 was significantly inhibited by the pre-treatment of HUVECs with a PAFR blocker. Our results further show that similar to the findings of Appanna et al [15], incubation of HUVECs with serum from dengue patients resulted in down regulation of ZO-1 expression but we here show that this was significantly inhibited by pre-treatment with a PAFR blocker. This suggests that PAF present at high concentrations in serum of dengue patients is likely to contribute to vascular leak by reducing expression of tight junction proteins. In addition to our experiments with HUVECs in assessing ZO-1 expression, we also investigated the effect of dengue serum on TEER in HUVECs. We found that serum from dengue patients significantly reduces the TEER in HUVECs when compared to serum from healthy individuals and this reduction of TEER was significantly inhibited if the HUVECs were pre-treated with a PAFR blocker prior to addition of serum from dengue patients. Therefore, these data further confirm that PAF present in serum of dengue patients reduces TEER in the endothelium, as this reduction was significantly ameliorated by a PAFR blocker. However, although the reduction of TEER in HUVECs was significantly inhibited by the use of a PAFR blocker, the TEER still did not return to normal, suggesting that apart from PAF, other mediators in the serum could also contribute to the vascular leak.

PAF is a potent inflammatory lipid mediator rapidly produced by many cells, such as endothelial cells, monocytes, mast cells and leucocytes following cellular stress [31]. It is known to cause hypotension, thrombocytopenia, increased vascular permeability and cardiac dysfunction when experimentally administered to animal models [32–34]. In dengue infection, platelets have been shown to be highly activated and platelet-monocyte aggregates have shown to contribute to the increase in vascular permeability [22]. Although there could be multiple pathways leading to activating of platelets, PAF could be further be contributing significantly to platelet activation and thus the immunopathology of the severe forms of the disease.

In this study we also found that PAF varied in the same patient in samples collected in the morning and the afternoon. Since we sampled patients only twice a day, it is difficult to comment if the variation in PAF levels was diurnal or whether such variations are observed more frequently. It has been shown that human monocytes produce PAF in a bi-phasic pattern when stimulated with LPS, which was shown to be due to the effects cytokines such as TNFα and IL-1β [31,35]. PAF has been shown to activate transcription of NF-κB resulting in expression of many inflammatory cytokines such as TNFα and IL-1β [31,35,36]. Since LPS was the main stimulus that resulted in bi-phasic production of PAF and other cytokines, it is possible that LPS plays a similar role in acute dengue infection. For instance, it has been shown that patients who develop plasma leakage have significantly higher levels of LPS than those who did not have plasma leakage [37]. Therefore, the possibility of LPS driving the production of PAF and other cytokines should be further investigated.

In summary, our results show that PAF levels were significantly higher in more severe forms of dengue and were associated with a reduced expression of tight junction proteins and reduced cell layer integrity that is likely to result in an increased paracellular leak. Use of PAFR blockers significantly reduced these effects; therefore, our results suggest the PAF is likely to be playing a significant role in inducing vascular leak in acute dengue infections; this has implications for the future management of patients such as use of PAFR blocker in acute dengue infection.

## Supporting Information

S1 checklist STROBE Checklist. (DOCX)
